# Development and validation of a pyroptosis-related prognostic signature associated with osteosarcoma metastasis and immune infiltration

**DOI:** 10.1097/MD.0000000000037642

**Published:** 2024-04-05

**Authors:** Zhenyu Gong, Yimo Wan, Enen Han, Xiaoyang Zhou, Jiaolong Huang, Hui Yu, Yihua Shi, Kai Lian

**Affiliations:** a Department of Orthopedics, Xiangyang No.1 People’s Hospital, Hubei University of Medicine, Xiangyang, China; b Key Laboratory of Zebrafish Modeling and Drug Screening for Human Diseases of Xiangyang City, Department of Obstetrics and Gynaecology, Xiangyang No. 1 People’s Hospital, Hubei University of Medicine, Xiangyang, China.

**Keywords:** chemotherapy, immune infiltration, osteosarcoma, prognostic analysis, pyroptosis

## Abstract

Pyroptosis is a programmed cell death, which has garnered increasing attention because it relates to the immune and therapy response. However, few studies focus on the application of pyroptosis-related genes (PRGs) in predicting osteosarcoma (OS) patients’ prognoses. In this study, the gene expression and clinical information of OS patients were downloaded from the Therapeutically Applicable Research to Generate Effective Treatments (TARGET) database. Based on these PRGs and unsupervised clustering analysis, all OS samples can be classified into 2 clusters. The 8 key differential expressions for PRGs (*LAG3, ITGAM, CCL2, TLR4, IL2RA, PTPRC, FCGR2B*, and *CD5*) were established through the univariate Cox regression and utilized to calculate the risk score of all samples. According to the 8-gene signature, OS samples can be divided into high and low-risk groups and correlation analysis can be performed using immune cell infiltration and immune checkpoints. Finally, we developed a nomogram to improve the PRG-predictive model in clinical application. We verified the predictive performance using receiver operating characteristic (ROC) and calibration curves. There were significant differences in survival, immune cell infiltration and immune checkpoints between the low and high-risk groups. A nomogram was developed with clinical indicators and the risk scores were effective in predicting the prognosis of patients with OS. In this study, a prognostic model was constructed based on 8 PRGs were proved to be independent prognostic factors of OS and associated with tumor immune microenvironment. These 8 prognostic genes were involved in OS development and may serve as new targets for developing therapeutic drugs.

## 1. Introduction

Osteosarcoma (OS) is a primary malignant tumor of the bone, with a bimodal distribution of onset age. The incidence rate is highest in children and adolescents (average age 18 years), the second (smaller) peak being in the elderly over 60 years old.^[1]^ OS has the characteristics of high malignancy and early distal metastasis, which is also the main reason for the poor prognosis and the 5-year survival rate of OS patients being <20%.^[2]^ Although the current surgical treatment, combined with neoadjuvant chemotherapy, has improved the survival rate of osteosarcoma patients, the prognosis of OS patients with recurrence or metastasis is still poor.^[3]^ Therefore, it is important to explore the prognostic genes and related therapeutic targets for improving the overall survival of OS patients.

Pyroptosis is a newly discovered mode of programmed cell death. It participates in the occurrence and development of a variety of diseases, promoting and inhibiting the formation of tumor and tumor micro-environments^[4]^ in particular. Pyroptosis is one of the latest hotspots in the research of antitumor drugs. On the one hand, pyroptosis can stimulate normal cells by releasing inflammatory factors and promote them to transform into tumor cells; On the other hand, these key inflammatory factors can promote tumor cell death and inhibit tumor cell proliferation and metastasis.^[5]^ In addition, previous studies have found that pyroptosis-related genes (PRGs) (*IRAK1, NOD2, POP1,* and *YWHAB*) can affect the prognosis of patients with hepatocellular carcinoma through immune function signaling pathways.^[6,7]^ PRGs (*AIM2, PLCG1, ELANE, PJVK, CASP3, CASP6,* and *GSDMA*) play an important role in tumor immunity in patients with ovarian cancer and can be used to predict its prognosis.^[8]^ However, the specific function of pyroptosis in the prognosis and treatment of OS is still in its infancy.

In this study, 8 PRG signatures were identified as having powerful prognostic functions in patients with OS and were verified in the GSE21257 cohort. Their differential expressions were strongly associated with survival rate and responses to immunotherapy. Our findings provide new evidence for exploring the prognostic biomarkers and therapeutic targets of OS patients.

## 2. Materials and methods

### 2.1. Data sources and processing

**Figure 1.**
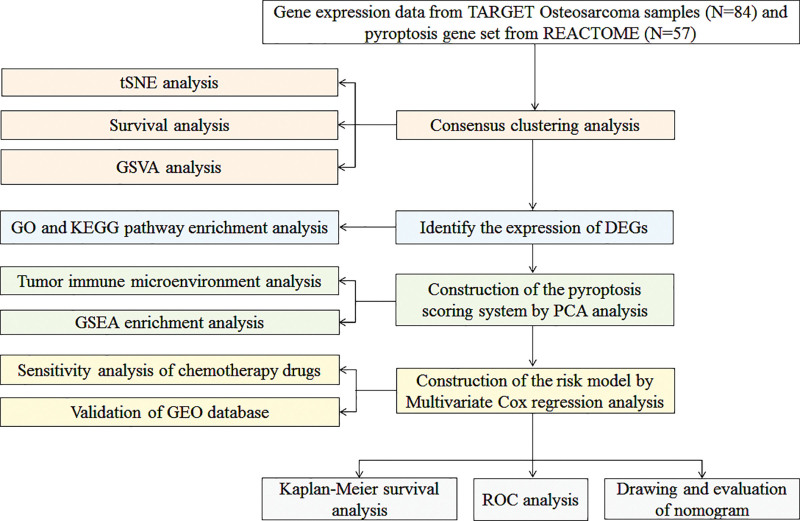
Flow chart of the study.

The workflow chart of this study is shown in Figure 1. The gene expression profiles of 84 OS samples were downloaded from the Therapeutically Applicable Research to Generate Effective Treatments (TARGET) (https://ocg.cancer.gov/programs/target) database. The external validation GSE cohorts and 57 OS samples were selected from gene expression omnibus (http://www.ncbi.nlm.nih.gov/geo) and named GSE21257. The OS RNA-seq was selected from TARGET as the training group and GSE21257 was used as the validation group.

### 2.2. Identification of pyroptosis-related differentially expressed genes (DEGs)

The PRGs in OS were obtained by intersecting 57 PRGs and they expressed genes in all 84 tumor samples. The differentially expressed PRGs in OS were screened by setting criteria with | log2 FC |> 1 and *P* < .05, using R package “DESeq2” software (version 1.32.0).

### 2.3. GO (gene ontology) and Kyoto encyclopedia of genes and genomes (KEGG) enrichment analysis

The DEGs were analyzed for enrichment using the R package ‘Cluster Profiler’ (version 4.0.2). Enrichment analysis referred to the GO, including a biological process, cellular component and molecular function. GO and KEGG terms, with the adjusted *P* < .05, were considered significantly enriched.

### 2.4. Construction and validation of pyroptosis-related gene signature

The univariate Cox regression analysis of 57 PRGs was performed by using the coxph function of survival in the R package and used to identify the PRGs for patients with OS in the training cohort. A threshold *P* < .05 was set to determine the prognostic variables. The 8 key PRGs were screened to construct the risk model, which was established by multivariate Cox regression analysis. A hazard ratio (HR) < 1 was a protective factor and, conversely, an HR ≥ 1 was a risk factor. The formula for the risk score of each sample was calculated as follows: Risk Score = (−0.07373 × LAG3 expression) + (0.62653 × ITGAM expression) - (0.13259 × CCL2 expression) - (0.1243 × TLR4 expression) - (1.15414 × IL2RA expression) - (0.3467 × PTPRC expression) - (0.73328 × FCGR2B expression) - (0.28154 × CD5 expression). The individual risk score of each sample was calculated and all samples were distributed into high- and low-risk score groups, according to the median of risk score. Kaplan–Meier (K-M) curves and receiver operating characteristic (ROC) scores were calculated to evaluate the prognostic value of the model, both in the training cohort and the test cohort.

### 2.5. Construction and validation of the nomogram

A predicated nomogram was developed by including clinical characteristics (age, gender, tumor stage, and T/N/M stages) and the risk score, which was used to predict the 1-, 3-, and 5-year overall survival. K-M, ROC and calibration curves of 1-, 3- and 5-year were used to evaluate the accuracy of the nomogram.

### 2.6. Estimation of immune cell infiltration and status related to pyroptosis

The immune score of each sample was calculated using the “ESTIMATE” package (version 1.1.0) and the fraction of immune cells between high- and low-risk score groups were assessed by the xCell algorithm using the immunedeconv package. The difference between high- and low-risk score groups was detected using a Wilcoxon rank-sum test. The expression of immune checkpoint-related genes were also detected by using the Student *t* test.

### 2.7. Statistical analysis

All of the analysis in this study was performed with R software (version 4.0.5), including the following packages: DESeq2, Limma, clusterProfiler, ESTIMATE, GEOquery, ggplot2, ggstatsplot, GOplot, GSVA, pheatmap, survivalROC, Rtsne, survival, survminer and VennDiagram. The Wilcoxon test was performed to analyze differences between the 2 groups. *P* < .05 was considered as being statistically significant.

## 3. Results

### 3.1. Identification of clusters based on PRGs expressed in OS

**Figure 2.**
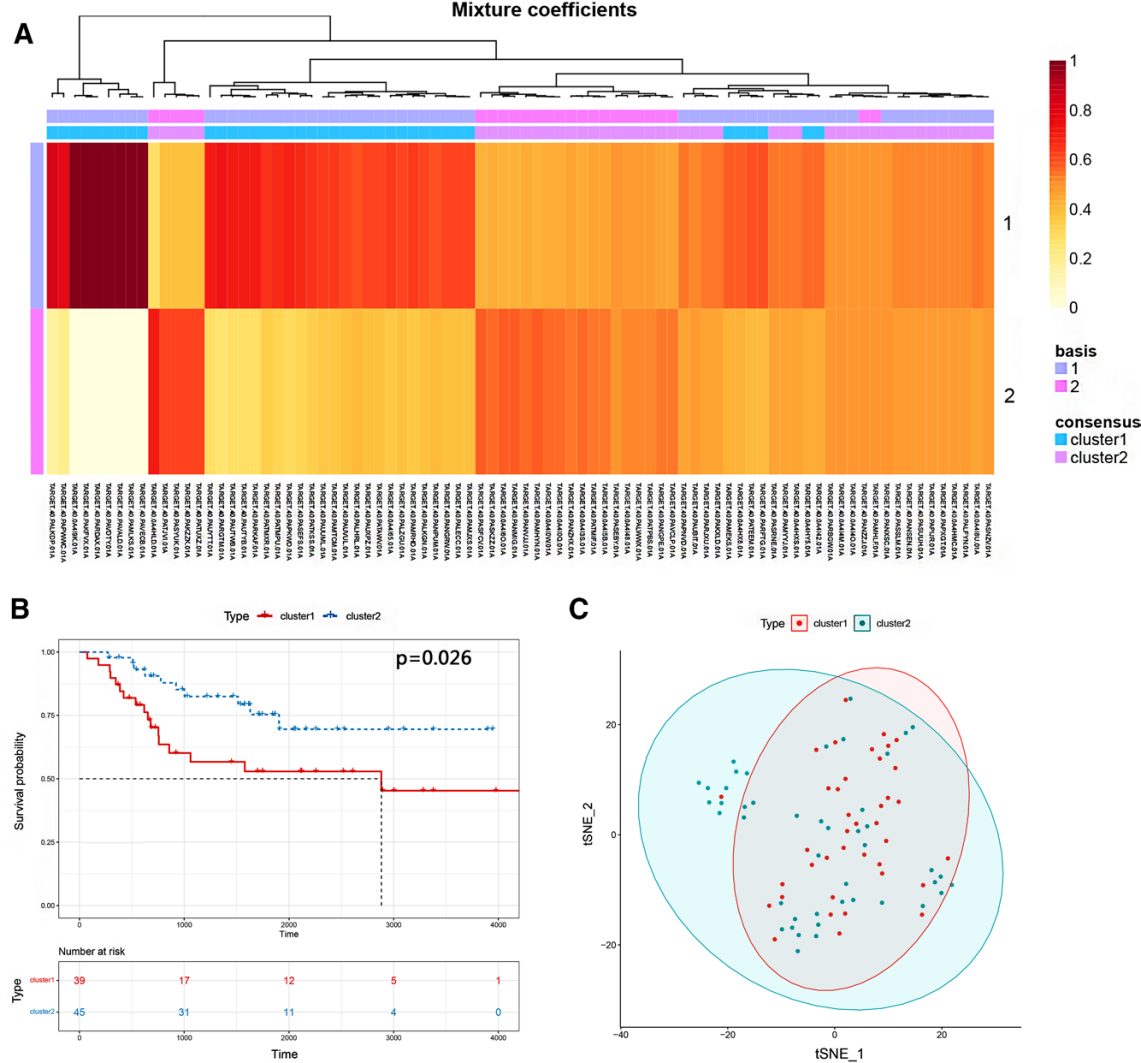
Expression of PRGs in osteosarcoma samples. (A) Heatmap of pyroptosis-related genes in osteosarcoma samples. (B) Kaplan–Meier survival analysis of osteosarcoma patients grouped into 2 clusters. (C) Venn diagram of pyroptosis-related genes in osteosarcoma samples. PRGs = pyroptosis-related genes.

Firstly, we obtained the mRNA expression data of 57 PRGs in 84 OS samples from the TARGET database. These samples were divided into 2 clusters after the unsupervised cluster analysis (Fig. 2A). The K-M curve revealed that the overall survival rate of cluster 2 is better than that of cluster 1 (Fig. 2B). The t-distributed stochastic neighbor embedding analysis was performed by combining the pyroptosis pattern of OS samples to visualize the resulting clusters by using the R package Rtsne (Fig. 2C).

### 3.2. The correlation between PRGs and tumor immune infiltrating

**Figure 3.**
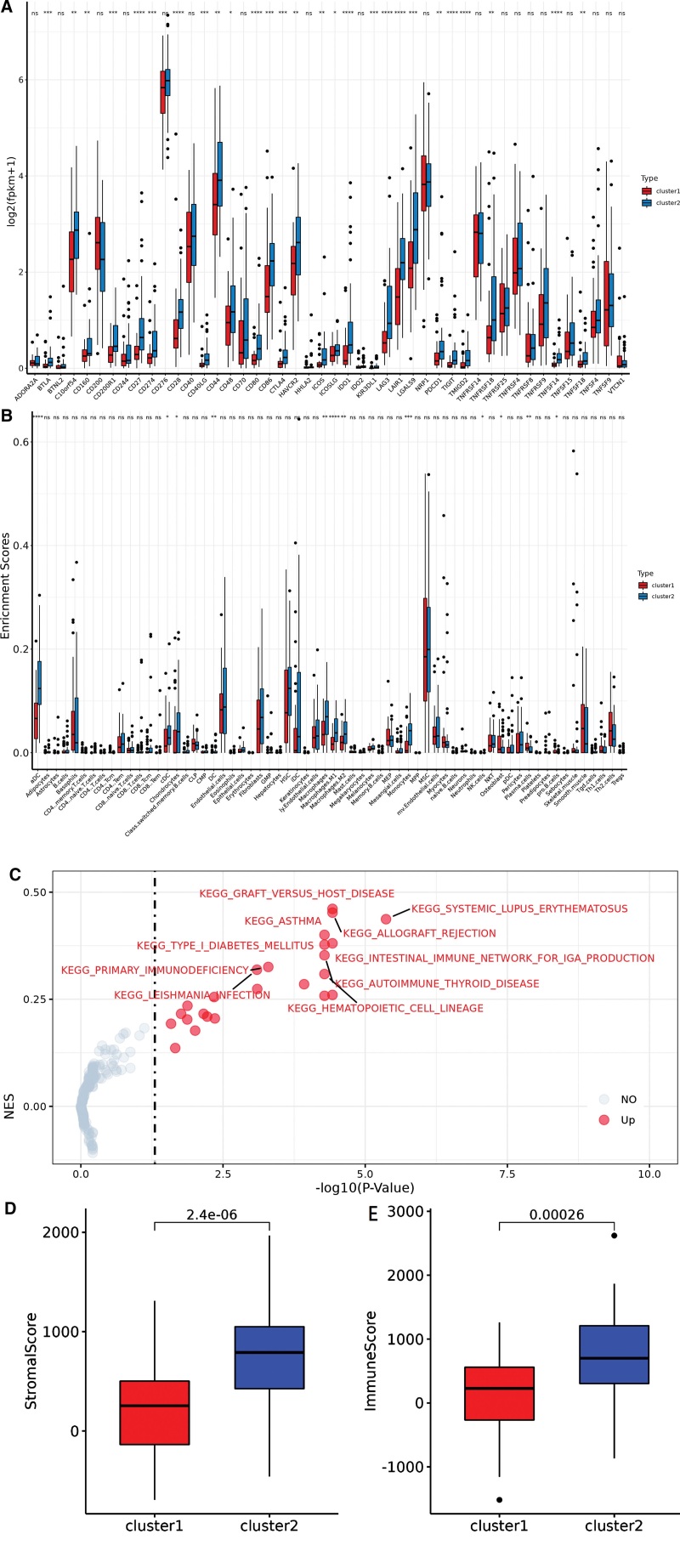
Comparison of immune cells and immune checkpoints in 2 clusters. (A, B) Difference of immune cells and immune checkpoints between 2 clusters. (C) GSVA enrichment pathway in pyroptosis mode of 84 osteosarcoma samples. (D, E) Boxplot of stromal and immune score between 2 clusters. GSVA = gene set variation analysis.

We investigated the immune status in 2 PRGs-based clusters and found that the expression of immune checkpoint-related genes, including CD27, CD28, CD80, IDO1, LAG3, LAIR1, TIGIT, TMIGD2 and TNFSF14, had significant differences between cluster1 and cluster2 (Fig. 3A, S1, http://links.lww.com/MD/L990). Moreover, the enrichment scores of aDC, macrophages M1 and monocytes were significantly different between the 2 clusters (Fig. 3B). We performed Gene Set Variation Analysis enrichment analysis to explore the variation in pathways in distinct pyroptosis patterns. As shown in Figure 3C, cluster 2 was significantly enriched in the upper pathways of graft-versus-host disease, systemic lupus erythematosus, allograft rejection, type I diabetes mellitus and intestinal immune network for IgA production. The stromal and immune scores of cluster2 were much higher than cluster1 (Fig. 3D–E).

### 3.3. Identification and enrichment analysis

**Figure 4.**
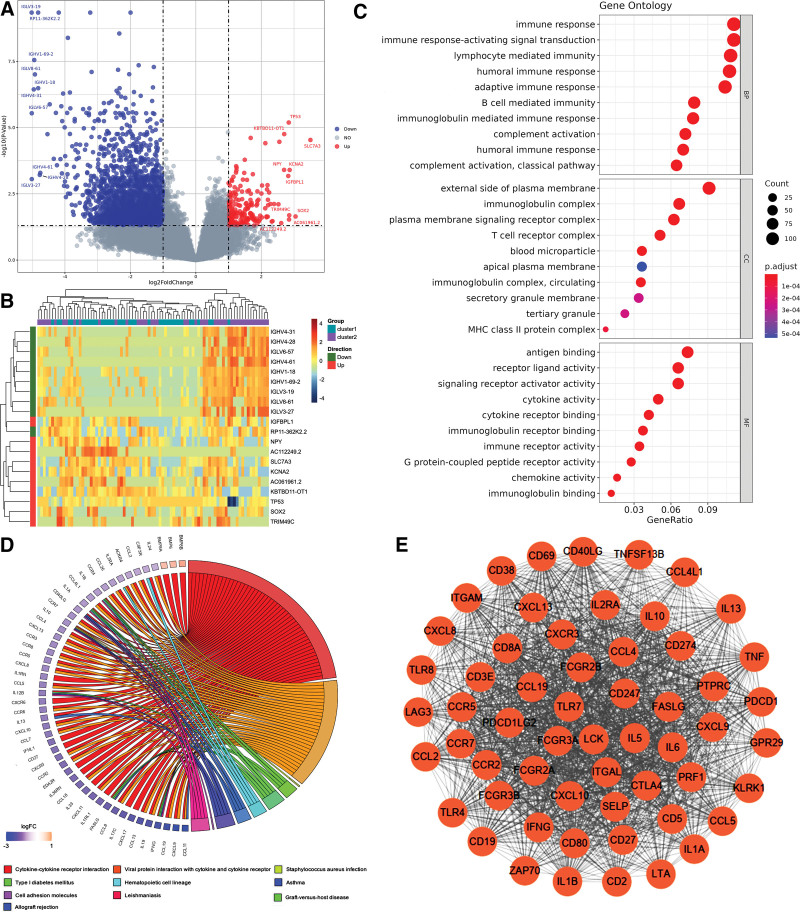
Functional enrichment analysis of pyroptosis-related DEGs. (A) Volcano plot of the DEGs in 2 clusters. Red indicates up-regulated gene, blue indicates down-regulated gene. (B) Heatmap of pyroptosis-related DEGs in OS samples. (C) The 558 pyroptosis-related DEGs of GO enrichment terms in BP, MF and CC. (D) KEGG enrichment analysis. (E) The PPI network of PRGs in OS. BP = biological process, CC = cellular component, DEGs = differentially expressed genes, GO = gene ontology, KEGG = Kyoto Encyclopedia of Genes and Genomes, MF = molecular function, PPI = protein interaction analysis, PRGs = pyroptosis-related genes.

The DESeq2 package was used to identify the 2 clusters of DEGs. The 206 up-regulated genes and 2636 down-regulated genes were obtained (Fig. 4A). GO enrichment analysis was performed on 558 DEGs and it was found that they were enriched in biological processes such as immune response-activated cell surface receptor signaling pathway, immune response-activated signal transduction, lymphocyte mediated immunity, humoral immune response and other immune-related processes (Fig. 4B). Furthermore, the KEGG enrichment analysis of the top 50 DEGs showed that the enriched pathways were mainly associated with cytokine-cytokine receptor interaction, and viral protein interaction with cytokines and cytokine receptors (Fig. 4C). Through the protein interaction analysis of all DEGs, we obtained the 57 most closely related genes as a follow-up study (Fig. 4D). In particular, 8 DEGs (including LAG3, ITGAM, CCL2, TLR4, IL2RA, PTPRC, FCGR2B and CD5) played important roles in the pyroptosis process (Fig. 4E).

### 3.4. OS patients with PCA score held better survival probability

**Figure 5.**
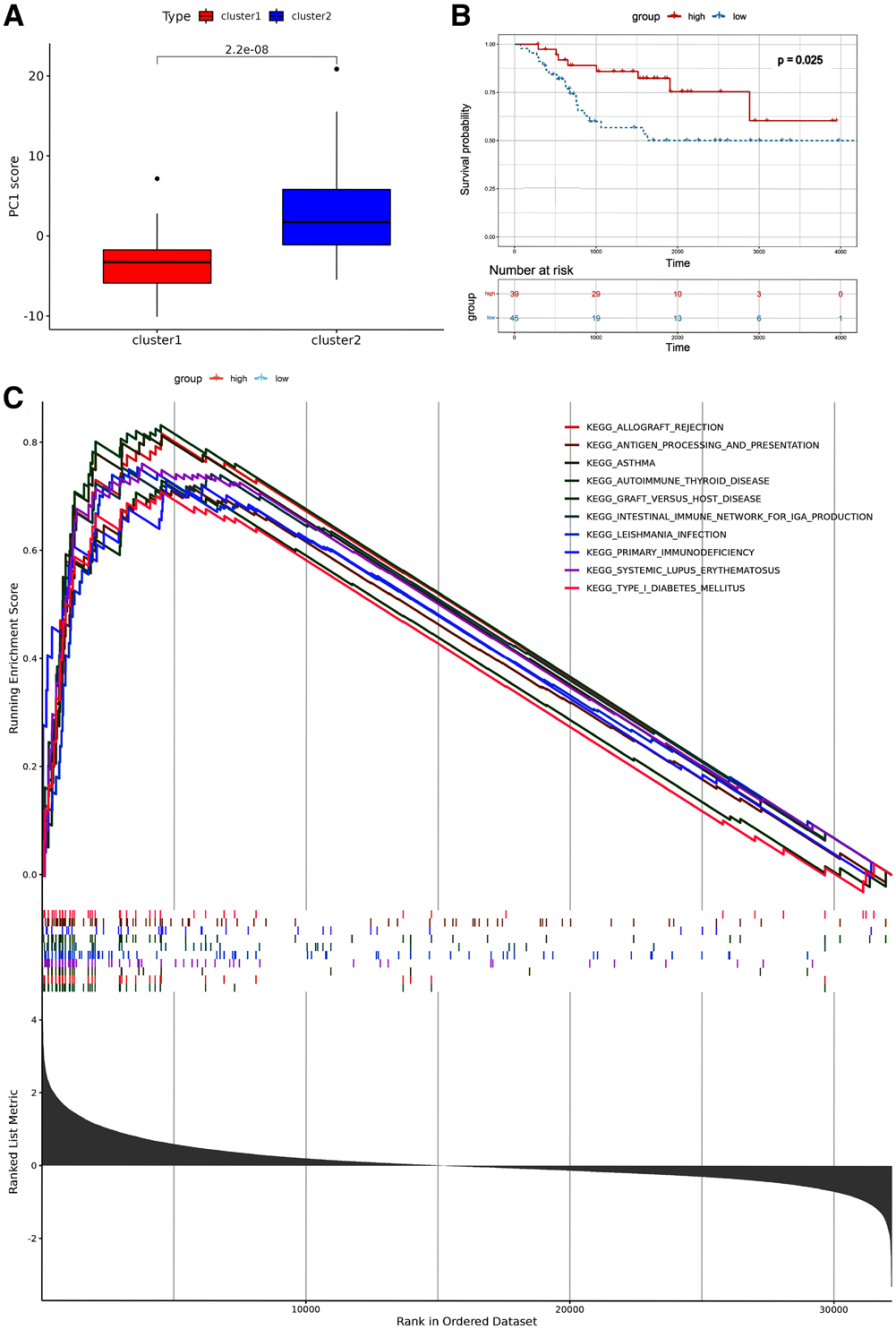
Survival analysis of pyroptosis-related genes in the TARGET cohort. (A) PCA plots of the TARGET cohort based on 57 pyroptosis-related genes. (B) Kaplan–Meier survival analysis of 2 clusters in the high- and low-risk groups. (C) GSEA for risk signature. GSEA = gene set enrichment analysis, PCA = principal component analysis, TARGET = therapeutically applicable research to generate effective treatments.

Based on the 57 genes most closely related to OS screened by protein-protein interactions, each sample was scored by Principal Component Analysis (PCA), and the samples were divided into a high expression group and a low expression group, according to the median value of the PCA score, the PCA score between the 2 clusters was statistically analyzed. We found that the PCA score of cluster2 was significantly higher than that of cluster1 (*P < *.001) (Fig. 5A). According to the PCA score and the survival of 84 OS samples, the K-M survival curve was plotted by using the ‘survminer’ package in R software. We found that the survival of the high expression group was higher than that of the low expression group (*P = *.025) (Fig. 5B). Gene set enrichment analysis depicted 10 correlated pathways, including intestinal immune network for IgA production, asthma, allograft rejection, and systemic lupus erythematosus, in both the high and low expression group (Fig. 5C).

### 3.5. Construction and validation of a PRGs-based prognostic model for OS patients

**Figure 6.**
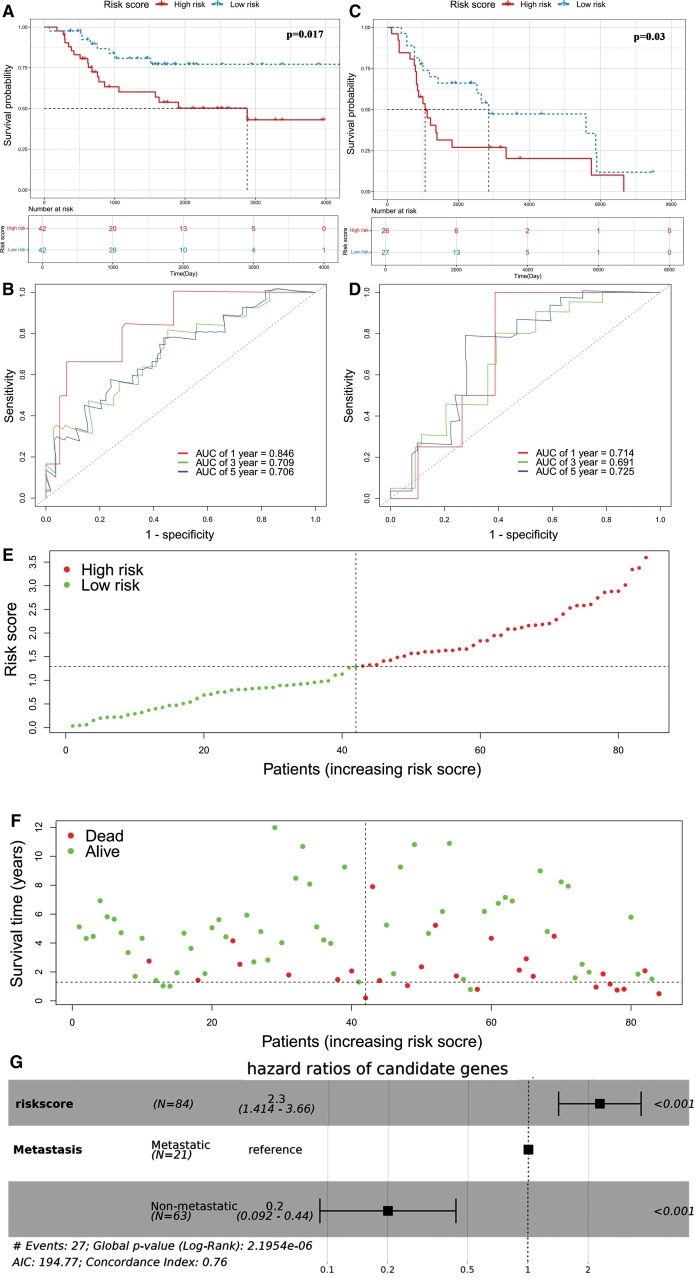
Construction and validation of the risk model in the TARGET-OS and GSE21257 cohort. (A,C) K-M curves for the OS of patients in the high and low-risk groups in training sets and test sets. (B,D) ROC curves demonstrated the predictive efficiency of the risk score in training sets and test sets. (E) The distribution and median value of risk score. (F) The scatter plot of patients associated with risk scores. (G) Multivariate independent prognostic analysis of risk score and clinical factors. K-M = Kaplan–Meier, ROC = receiver operating characteristic, TARGET = therapeutically applicable research to generate effective treatments.

In accordance with the coefficient value of the 8 PRGs, the risk scores of all samples were calculated and ranked in the training set and the validation set. Based on the risk score, patients in the training set were divided into the high-risk and low-risk groups. As shown in Figure 6A, OS patients with a high-risk score showed a worse survival time than patients with low-risk score in the training set (*P = *.017), which was used to construct a PRGs-based prognosis model. ROC curves revealed that the area under the ROC curve (AUC) values of 1-, 3-, and 5-year in the training test were 0.846, 0.709, and 0.706 (Fig. 6B). These results indicated that the risk model was valid for predicting prognosis for OS patients. The validation set K-M survival curve showed that the survival rate of the high-risk group in the validation set was much lower than that of the low-risk group (*P = *.030) (Fig. 6C).

According to the risk model constructed above, the risk score of the samples in the validation set was calculated based on the survival of the samples in the validation set and the expression levels of the 8 prognosis-related DEGs (Table 1, S2, http://links.lww.com/MD/L991). According to the ROC curve of the validation set, the AUC of 1-, 3- and 5-year were 0.714, 0.691 and 0.725, respectively, indicating that the constructed risk model can better predict the 1-, 3- and 5-year overall survival rates of OS patients in the validation set (Fig. 6D).

**Table 1:** Univariate cox regression analyses of the pyroptosis-related DEGs.

Gene	HR	HR.95L	HR.95H	*P* value
LAG3	0.447437	0.207695	0.963914	.039993
ITGAM	0.504261	0.290736	0.874604	.014816
CCL2	0.630664	0.425076	0.935684	.022008
TLR4	0.601461	0.37794	0.957177	.031986
IL2RA	0.260854	0.084072	0.809368	.020015
PTPRC	0.608971	0.377313	0.982859	.04228
FCGR2B	0.372424	0.146222	0.948556	.038388
CD5	0.222929	0.052203	0.951993	.042725

DEGs = differentially expressed genes.

In the training set, the median risk score was used as the cutoff value and the patients were divided into high-risk (n = 42) and low-risk (n = 42); then, the risk curves were drawn from low to high (Fig. 6E). Figure 6F shows the distribution map of survival status of the patients. The patient survival time obviously decreases with the increase in the patient risk value (Fig. 6F). Furthermore, in a multivariate Cox analysis of the PRGs-based risk score and clinical features, the results indicated that the risk score and metastasis at diagnosis were independent prognosis factors for patients with OS (Fig. 6G).

### 3.6. Pyroptosis risk signature predicts chemotherapy sensitivity

**Figure 7.**
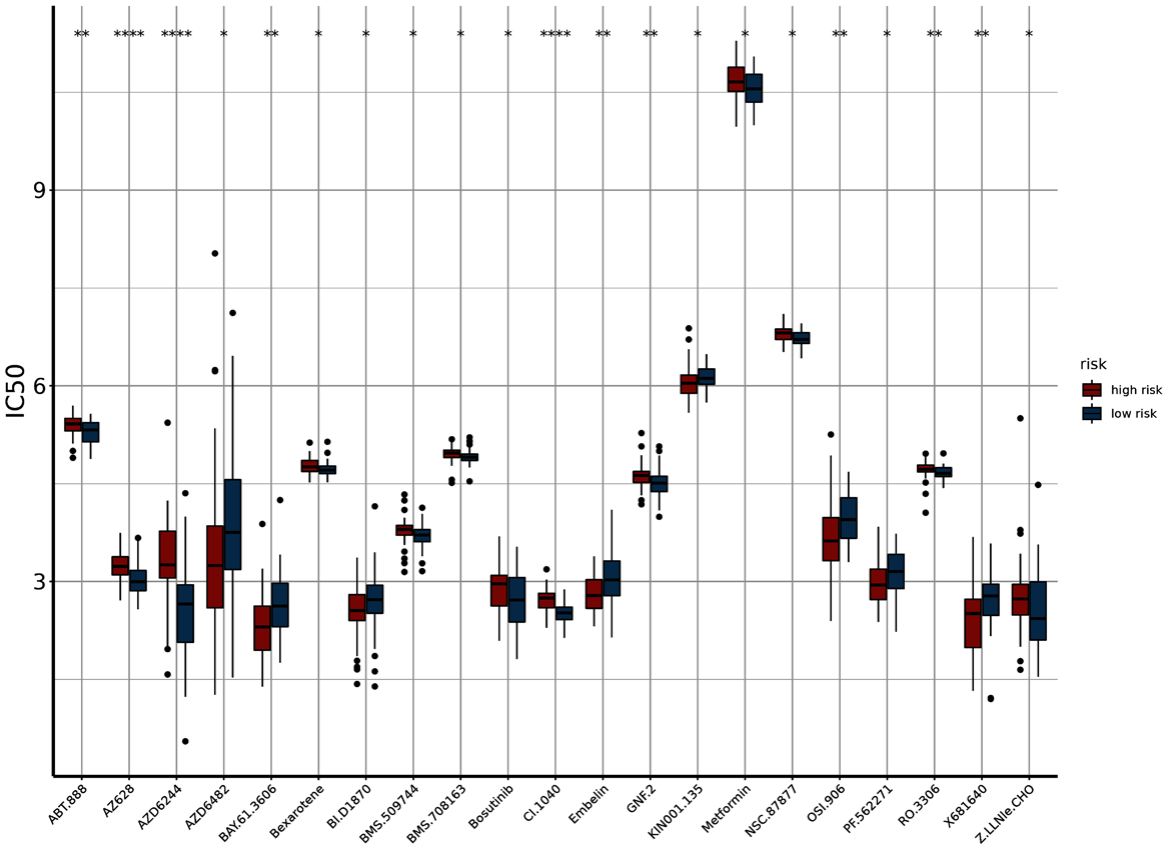
Difference of IC50 between high- and low-risk groups. IC50 = half maximal inhibitory concentration.

We then investigated the correlation between risk score and drugs sensitivity using the pRRophetic R package, based on the Genomic of Drugs Sensitivity in Cancer database. The results indicated high-risk scores associated with 11 chemotherapy drugs, including AZ628, AZD6244 and CI-1040 (*P < *.0001) (Fig. 7).

### 3.7. Constructing a nomogram based on risk scores and clinical information

**Figure 8.**
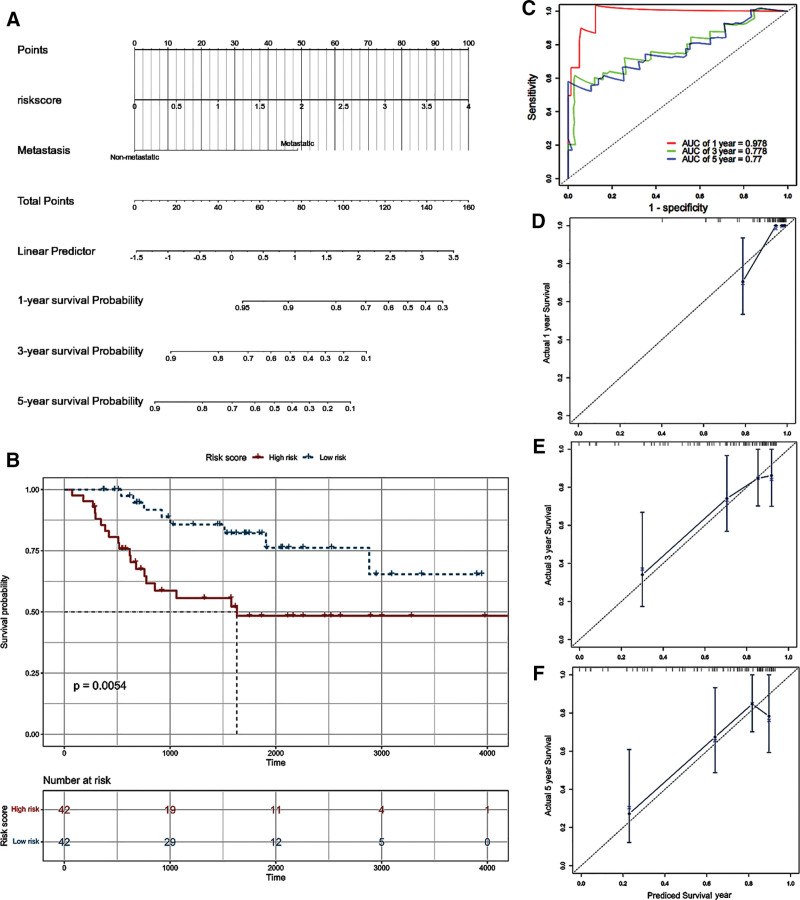
Construction and evaluation of a nomogram. (A) Nomogram to predict survival of patients with osteosarcoma. (B) Kaplan–Meier survival analysis of osteosarcoma in the low and high-risk groups. (C) The ROC curves of the nomogram with regard to 1-, 3-, and 5-yr survival of osteosarcoma patients. (D-F) The calibration curves of the nomogram. ROC = receiver operating characteristic.

To determine the evaluation of a PRGs-based prognosis model and clinical features in the prognosis of OS patients, we built a nomogram that combined the PRGs-based prognosis model with the clinical features, such as risk score and metastasis. A nomogram integrating the risk score and metastasis was established to predict OS prognosis (Fig. 8A). The K-M curve displayed the correlations between the risk model and the prognosis of OS patients, demonstrating that the survival probability of patients in the low-risk group was higher than that of the high-risk group (*P* < .01) (Fig. 8B). Considering the clinical characteristics, the ROC curve analysis showed that the AUC for OS at 1-, 3-, and 5-year was 0.975, 0.776, and 0.770, respectively (Fig. 8C). The calibration plots for 1-, 3-, and 5-year also revealed that there is a good consistency between the predicted survival probability and the actual observed results (Fig. 8D–F).

## 4. Discussion

OS was one of the highly aggressive tumors which frequently developed metastasis. Although chemotherapy and surgery treatments have improved the survival of OS patients, the survival rate of patients with recurrent or metastatic OS has not been improved in the last 30 years. Pyroptosis is a kind of programmed cell death, which plays a dual role in promoting and inhibiting the growth of hepatocellular carcinoma, breast carcinoma and renal cell carcinoma, as well as other cancers.^[9–11]^ Pyroptosis has been proved to be related to tumor proliferation and metastasis, it is closely related to tumor microenvironment and tumor immunity. In recent years, several studies have shown that pyroptosis enhances antitumor immunity in cancer treatment and becomes an effective cancer treatment.^[12–14]^ It is necessary to find better treatment and prognosis prediction for OS patients. Despite the fact that recent advances have demonstrated the regulatory effect of PRGs on a genetic and transcriptional level for OS, the global alterations in PRGs have not been characterized at metastasis in OS.

In this study, 8 key pyroptosis genes, namely LAG3, ITGAM, CCL2, TLR4, IL2RA, PTPRC, FCGR2B and CD5, were identified as signature genes and could predict the prognosis in patients with OS. We have constructed and validated a prognosis model based on 8 pyroptosis-related DEGs, to more accurately predict the prognosis of OS patients. TLR4 and ITGAM were strongly associated with macrophage polarization in the tumor microenvironment.^[15]^ The increased expression of ITGAM can decrease pulmonary inflammation and disruption of the endothelial cell barrier.^[16]^ ITGAM was identified as a serum exosomal protein marker of lung adenocarcinoma.^[17]^ ITGAM might be strongly linked to the carcinogenesis and the development of anaplastic thyroid cancer.^[18]^ The CCL2-CCR2 axis exerts 85 significant biological activities in dogs with metastatic OS.^[19]^ Inhibiting the TLR4 pathway of macrophages could achieve tumor inhibition.^[20]^ TLR4 activation may suppress the progression of OS via stimulating CD8 + cells.^[21]^ The antagonist LAG3 increased survival and antitumor immunity.^[22]^ The expression levels of PTPRC attenuates tumor development.^[23]^ Exogenous CD5 inhibits cell proliferation of hepatocellular carcinoma.^[16]^ These results suggested that 8 key pyroptosis genes were related to the progression of the respective cancers and the alteration of the tumor microenvironment. Although 8 key pyroptosis genes have made some progress in the development of some diseases, there is little research on OS. In this study, the prognosis model was constructed by combining risk score and metastasis to estimate the prognosis of patients, which showed good predictive performance and accuracy by drawing the calibration curve. In the future, we believe that the pyroptosis gene signature in this research was a convincible prognosis biomarker and could be applied in clinics.

Pyroptosis has been reported to occur with a strong inflammatory response, which is activated by inflammasomes, such as a NLR family pyrin domain containing 1 (NLRP1).^[24]^ A number of studies have shown that innate immune cells contribute to OS suppression through direct recognition, apoptosis, and the adaptive immune response.^[25]^ Previous investigations showed that the mutation of ITGAM and TLR4 is closely related to OS.^[15]^ CCL2 was identified as pivotal prognostic signatures, which could guide the clinical decision making in the treatment of OS.^[26]^ In the present study, we identified the overlapped differentially expressed mRNAs (DEmRNAs) related to the pyroptosis-related prognostic signature in the TARGET and GSE21257 Datasets. The functional analyses of these differentially expressed messenger RNAs showed that they were enriched in several immune-associated functions and pathways, such as immune response-activating cell surface receptor signaling pathway, immune response-activating signal transduction, lymphocyte mediated immunity and humoral immune response. Our results further demonstrated that the 8 pyroptosis-related genes were possibly involved in immune-related pathways and the modulation of immunity, suggesting that pyroptosis may be related to the improvement of OS prognosis.

In this study, the risk model based on pyroptosis-related genes can more accurately predict the survival rate of patients with OS. In addition, we studied the predictive ability of 138 kinds of chemotherapy-targeted therapy drugs and the results clearly distinguished the chemotherapy-targeted therapy drugs applicable to high-risk groups, providing a certain reference for the precise treatment of OS patients. Previous studies have demonstrated that the aDC controlled tumor growth and stimulated anticancer immune responses.^[27]^ Beneficial M2-to-M1 polarization of macrophages could induce pyroptosis to enhance antitumor immunity.^[28]^ At present, our research further fills the gap of a prediction model based on pyroptosis-related genes. Of course, this study inevitably has some limitations. Firstly, due to the inherent characteristics of OS, the sample size of the validation queue is relatively small. Secondly, there is a lack of experimental work and the molecular mechanism of its specific participation still needs to be further studied.

In summary, we constructed and validated a pyroptosis-related prognostic model associated with OS metastasis and risk score. The lower the risk score of this prognostic model, the better the effect of immunotherapy. Its stability and accuracy were further assessed. The pyroptosis state of cancer cells could serve as the prognostic biomarker and inspire new ideas for pyroptosis or immune-targeted therapy. These 8 prognostic pyroptosis-associated genes emphasized PRGs’ critical clinical significance and provide fresh ideas for directing individualized chemotherapy and immunotherapy for OS patients. More basic research and clinical research should be carried out to confirm our findings and clarify the fundamental mechanisms of pyroptosis in OS. Hence, further functional experimental research is warranted in the future.

## Author contributions

**Conceptualization:** Zhenyu Gong, Yimo Wan, Enen Han.

**Data curation:** Zhenyu Gong.

**Formal analysis:** Zhenyu Gong, Yimo Wan.

**Funding acquisition:** Jiaolong Huang, Hui Yu.

**Investigation:** Zhenyu Gong.

**Methodology:** Zhenyu Gong, Xiaoyang Zhou.

**Resources:** Yihua Shi.

Software: Enen Han, Jiaolong Huang.

**Supervision:** Yihua Shi, Kai Lian.

**Visualization:** Hui Yu.

**Writing – original draft:** Zhenyu Gong, Yimo Wan.

**Writing – review & editing:** Yihua Shi, Kai Lian.

## Supplementary Material




