# Supplementary figures and images for: Development and validation of a pyroptosis-related prognostic signature associated with osteosarcoma metastasis and immune infiltration
Source: Medicine (Baltimore). 2024 Apr 5;103(14):e37642. doi: 10.1097/MD.0000000000037642 (PMC10994441; doi:10.1097/MD.0000000000037642)

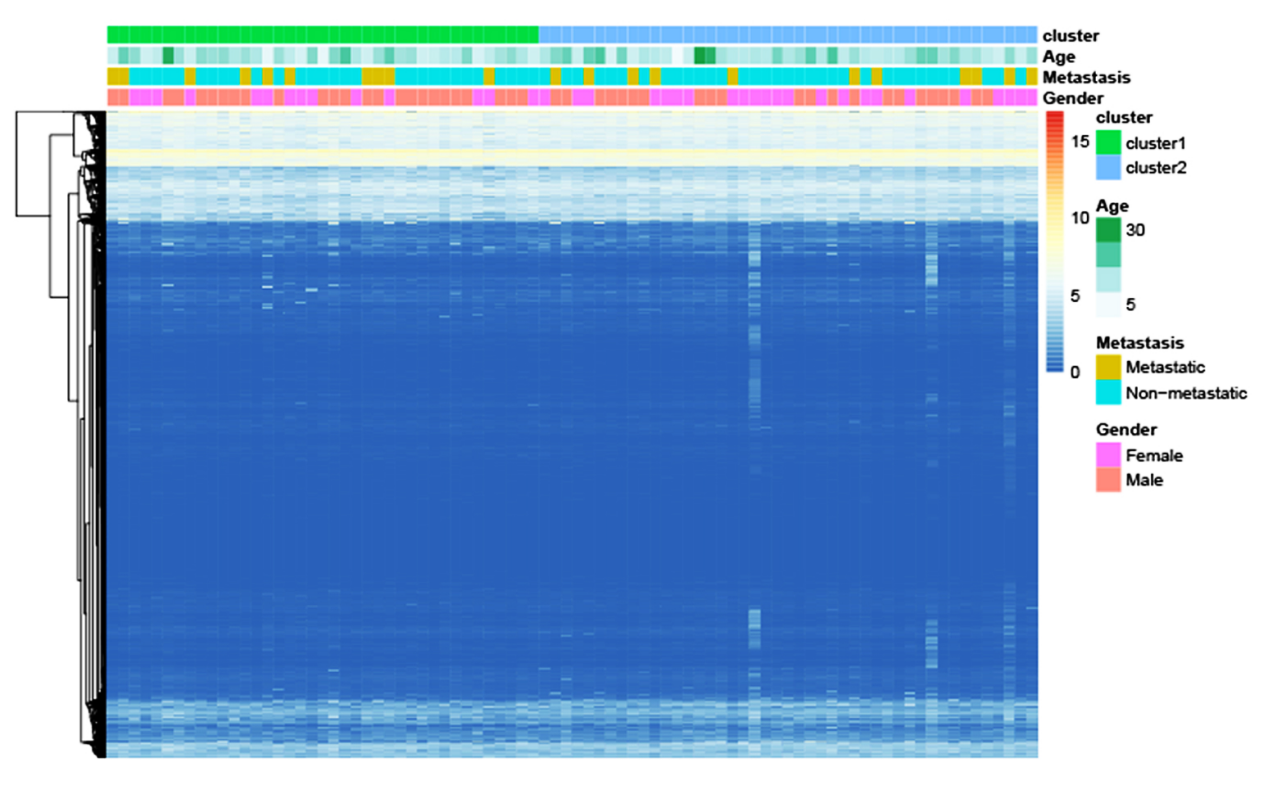


**Figure S1.** Heatmap of 84 samples gene expression.

Supplement: Supplementary file 1 [file medi-103-e37642-s001.docx]
