# Supplementary material for: Development and validation of a pyroptosis-related prognostic signature associated with osteosarcoma metastasis and immune infiltration
Source: Medicine (Baltimore). 2024 Apr 5;103(14):e37642. doi: 10.1097/MD.0000000000037642 (PMC10994441; doi:10.1097/MD.0000000000037642)
Supplement: Supplementary file 2 [file medi-103-e37642-s002.docx]

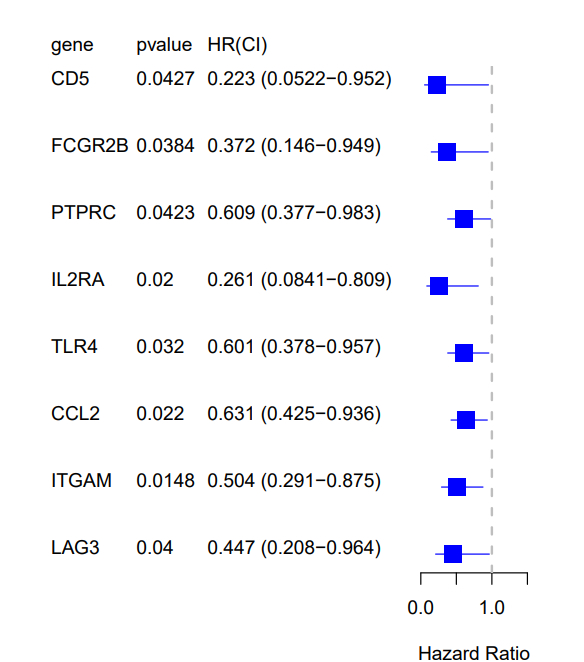


**Figure S2.** Univariate Cox regression analysis of overall survival for each pyroptosis-related DEG.
